# Post-COVID-19 Assessment of Physical, Psychological, and Socio-Economic Impact on a General Population of Patients From Odisha, India

**DOI:** 10.7759/cureus.30636

**Published:** 2022-10-24

**Authors:** Pratima Singh, Bidhu K Mohanti, Sangram Keshari Mohapatra, Akash Deep, Burgula Harsha, Mona Pathak, Shubhransu Patro

**Affiliations:** 1 Pulmonary Medicine, Kalinga Institute of Medical Sciences, Bhubaneswar, IND; 2 Radiation Oncology, Kalinga Institute of Medical Sciences, Bhubaneswar, IND; 3 General Medicine, Kalinga Institute of Medical Sciences, Bhubaneswar, IND; 4 Research and Development, Kalinga Institute of Medical Sciences, Bhubaneswar, IND

**Keywords:** outcomes research, economic impact, symptom burden, post-covid clinic, india, post-covid-19 conditions(pcc)

## Abstract

Aim: This prospective cross-sectional study evaluated the physical, psychological, and socioeconomic impacts of post-COVID-19 conditions (PCC) in a generalized population from Odisha, India.

Materials and methods: The study protocol and clinical record form (CRF) were approved by the Institutional Ethics Committee. Those above 18 years and of all genders who had recovered in the last six months, whether hospitalized or not hospitalized after the COVID-19 diagnosis, were included in our study.

Results: A total of 198 persons with a median age of 41 years (18-87 years) were enrolled at the post-Covid clinic. For COVID-19 management, 91 persons (46%) were hospitalized, and the remaining 107 (54%) were non-hospitalized. Five dominant clusters of physical symptoms were present - fatigue (82.8%), cough (54%), breathing difficulty (54%), pain in the body (53%), and sleeplessness (51%). The psychological issues faced were fear (41.6%), worry (40.4%), depression (31.8%), and anger (30.3%). The median monthly income in Indian Rupees (INR) for pre-Covid versus post-Covid was 30,000 versus 25,000, effectively a loss of 16.6% in the family income. Adverse impacts on health and economic conditions were observed in 31.3% and 20.7%, respectively.

Conclusion: Post-Covid clinics can be a resource-appropriate health system approach for nearly 20% of the pandemic survivors with a low gross domestic product (GDP) per capita.

## Introduction

COVID-19, caused by severe acute respiratory syndrome coronavirus-2 (SARS-CoV-2), has affected 530 million people worldwide with 6.3 million deaths to date. India, with a current population of 1.4 billion, has also been severely affected recording over 43 million cases and 0.52 million deaths [1]. Over the last two years, healthcare systems around the world have learned to take care of infected patients (hospitalized or non-hospitalized) and to prevent transmission by using appropriate measures and vaccination.

However, a new dimension of the COVID-19 disease has gradually emerged. At the start, COVID-19 was considered as an acute viral infection with an expected recovery within a few weeks. Being a novel disease, its trajectory in convalescent patients was unforeseen. From the initial reports of symptom burden at the post-recovery phase [2] to the present knowledge of long COVID as a heterogeneous and complex disease spectrum lasting for 12 weeks or beyond, this global pandemic has continued to impact the health systems [3]. With the increase in the number of infected patients, the number of survivors has expanded; the current case fatality rate (CFR) is at 1.2% [1]. Approximately 10% to 35% of those infected by and recovered from COVID-19 develop persistent or new symptoms lasting weeks or months. Although there is interchangeable use of terms such as “long COVID” or “long-haul COVID”, the WHO has labeled a consensus definition for adults - post-COVID-19 condition occurs in individuals with a history of probable or confirmed SARS-CoV-2 infection, usually three months from the onset, with symptoms that last for at least two months and cannot be explained by an alternative diagnosis. Whereas, the Centers for Disease Control and Prevention (CDC) in the USA has termed it as post-COVID conditions (PCC) which can have a wide range of symptoms that can last more than four weeks or even months after infection [3-5].

The pathophysiology of the post-COVID-19 state is a complex interplay of several factors, namely a persistent hyper-inflammatory state, inadequate antibody response, and ongoing viral activity. All these lead to the complications of a hyper-coagulable state, immunologic aberrations, inflammatory changes, and maladaptation of the angiotensin-converting enzyme 2 (ACE-2) pathway causing multi-system damages [6]. People with PCC may experience health problems from different types and combinations of symptoms happening over different lengths of time; per our present understanding, there is no test to diagnose PCCs [4,5]. Although fever, respiratory difficulty, and loss of taste are the dominant triad of a clinical picture in the acute infective phase, in the post-recovery phase, these may vary from general symptoms of fatigue and malaise to a combination of respiratory, cardiac, digestive, and psycho-neurological disorders which impact daily life [3-6].

There is substantial interest to study the long COVID or PCC outcomes in localized regions, as it may lead to better allocation of healthcare resources and more effective disease management measures for those regions hit by the pandemic. These findings may inform providers in emergency departments or critical care settings of treatment priorities, empower healthcare stakeholders with effective disease management strategies, and aid health policymakers in optimizing allocations of medical resources [7].

The first case of the COVID-19 pandemic was confirmed in the Indian state of Odisha on March 16, 2020, when a student returned from Italy with the disease but subsequently recovered. For a population of approximately 46 million in this state in Eastern India, there was a total of 12,88,576 confirmed COVID-19 positive cases out of which 12,79,270 recovered and 9,126 died [8]. This prospective study aims to report on the post-COVID patient population in Odisha, such that it will be able to provide the required knowledge to health system providers and policymakers. 

## Materials and methods

This was a prospective cross-sectional study approved by the Institutional Ethics Committee of the Kalinga Institute of Medical Sciences (KIMS), Bhubaneswar (Approval no: KIIT/KIMS/IEC/573/2021). After the first few COVID-19 confirmed cases were diagnosed in Odisha, a dedicated COVID hospital started functioning at KIMS on April 5, 2020. A total of 6,003 patients were admitted in the first wave (April 2020 to January 2021), out of whom 589 died, and 5414 were discharged. Whereas in the second wave (from March 2021 to November 2021), 3,007 patients were admitted, out of which 556 died, and the remaining 2,451 were discharged. On observing that many patients returned to the hospital in the post-COVID-19 recovery period with several ailments, a post-Covid clinic was established at KIMS.

This cross-sectional study was carried out at the post-covid clinic of KIMS, under Kalinga Institute of Industrial Technology (KIIT) Deemed to be University, Bhubaneswar from February 10, 2021 to June 10, 2021. The primary objective of the study was to identify the immediate physical issues faced by patients in the post-COVID-19 recovery period. The secondary objectives were to (a) observe the psychosocial effects in post-COVID-19 patients and (b) analyze the comorbidity and sociodemographic factors within the studied group. Accordingly, a study protocol and clinical record form (CRF) were designed by the investigators in-house and duly approved by the IEC, before the study was initiated. The records and data related to this study were maintained confidentially.

The study population for this cross-sectional study comprised all COVID-19-positive patients diagnosed with RTPCR or rapid antigen test or both; patients who had recovered in the last six months and above 18 years of age; patients of all genders; those who had either been hospitalized or had been treated at home in isolation per Odisha state guidelines; those with persistent symptoms for at least three months or more after COVID-19 diagnosis [4,5]. Informed consent was obtained from all the participants in the study.

Exclusion criteria included patients who were unable to provide COVID-19 positive information, those unable to comprehend the cross-sectional survey components (in the CRF), and those who did not agree to participate in this survey.

The demographic data like age, sex, and address were recorded. The socioeconomic condition of the patient before and after the COVID-19 infection was also recorded. The physical and psychological symptoms experienced in their post-Covid state were noted, along with the existing co-morbid conditions. The severity of physical and psychological symptoms was assessed on the self-report Likert scale of “Not at All”, “A Little”, “Quite a Bit”, and “Very Much”; as subjective responses from each of the patients on the 4-points scale [9]. The overall impact of COVID-19 in terms of Family Condition, Economic Condition, Health Condition, and Mental Condition were similarly noted on the self-report Likert scale - “As before”, “Affected a little”, “Can Manage”, and “Require Help”. The variable responses related to the severity of physical and psychological symptoms on the Likert scale such as “Quite a Bit” and “Very Much” were considered as requiring medical attention.

All the continuous parameters are described with mean± standard deviation or median (interquartile range) depending on the distribution of variables. All the categorical variables are reported as frequency (%) and in suitable graphs. The data were analyzed using Stata, version 15.1 (StataCorp, TX, USA) and Microsoft Excel 2016.

## Results

In this prospective study, a total of 198 persons were enrolled with PCC status, after the first and second wave of COVID-19 in Odisha state. At a median age of 41 years (range: 18 to 87 years), there were more males (67.2%) than females (34.8%). The diagnosis of COVID-19 infection by RT-PCR test was utilized more often (67.2%), compared to the rapid antigen test (18.7%) or, by both tests (14.1%). In this PCC cohort, 91 out of 198 (46%) were admitted to the hospital for a median hospital stay of seven days (range,0-78 days). The remaining 107 persons had a history of being treated either at a government-run COVID-19 care facility (8.1%) or were managed at home in isolation (46%). In both phases, medical care under the doctor’s supervision was provided to 185 (93.4%); while 11 (5.6%) in this cross-sectional survey stated that they had not got any supervised treatment. Overall, the acute phase of COVID-19 infection required a median treatment time of 14 days (range,0-70 days); 17 (8.6%) of the total patients required oxygen therapy for recovery. Details of the patient characteristics and COVID-19 management profile are given in Table 1. In this study, 193 out of 198 (97.5%) persons with PCC status had given consent to contact them by mobile phone.

**Table 1:** Patient Characteristics and COVID-19 Management Profile

Characteristic	Patients (n)	Percentage (%)
Post-Covid-19 Patients surveyed	198	-
Gender		
Female	69	34.8
Male	129	65.2
Age		
Median Years	41	-
Range	18-87	-
Covid-19 Test		
RT-PCR	133	67.2
Rapid Ag	37	18.7
Both	28	14.1
Covid-19 Treatment		
Home	91	46.0
Hospital	91	46.0
Covid-19 Facility	16	8.0
Treatment under Doctor’s Supervision		
Yes	185	93.4
No	11	5.6
Cannot Say	2	1.0
Treatment Days		
Median	14	-
Range	0-70	-
Hospital Stay Days		
Median	7	-
Range	0-78	-
Needed Oxygen		
Yes	17	8.6
No	181	91.4
Can be contacted by Mobile		
Yes	193	97.5
No	5	2.5

**Figure 1:**
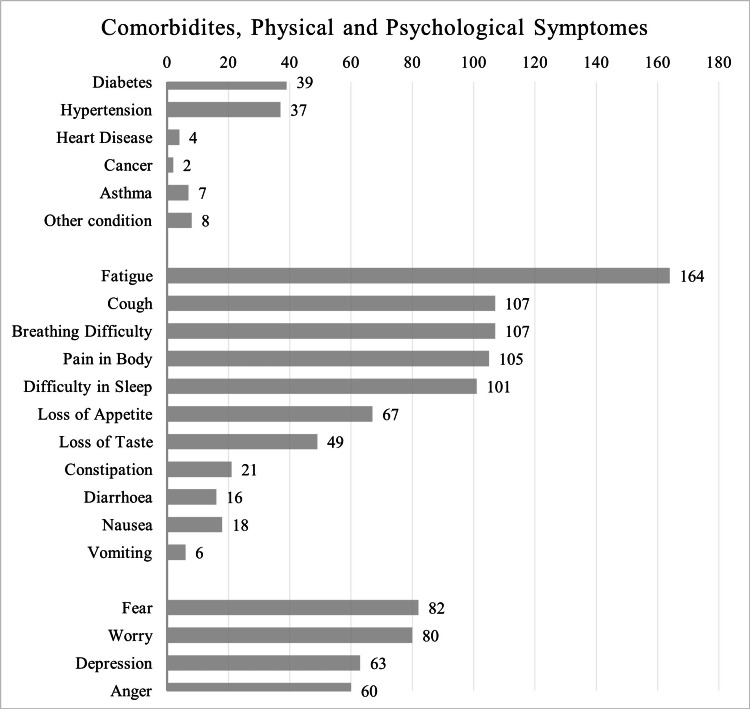
Comorbidities, Physical and Psychological Symptoms

**Figure 2:**
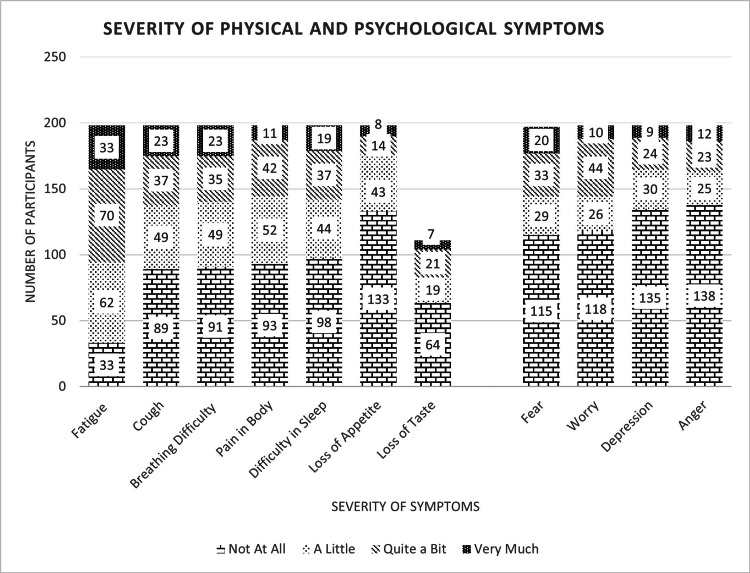
Severity of Physical and Psychological Symptoms

The clinical spectrum of this cohort was assessed at a median of 37 days after COVID-19 diagnosis (minimum 14 days, maximum 141 days). This evaluation recorded the co-morbidities, and physical and psychological symptoms (Figure 1). Diabetes mellitus and hypertension co-existed in 19.7% and 18.7% of patients, respectively. Fatigue (82.8%), cough (54%), breathing difficulty (54%), pain in the body (53%), and sleeplessness (51%) were the five dominant clusters of physical symptoms in more than half the studied cohort. In the PCC state, the psychological issues faced were fear, worry, depression, and anger by 41.6%, 40.4%, 31.8%, and 30.3% of the patients, respectively. The severity of this symptom burden on the Likert scale was recorded as “Quite a Bit” and “Very Much”, which implied a significant impact on daily living. They were analyzed together for the cohort of patients (n=198). The severity of symptoms were (a) physical symptoms: fatigue in 52.1%, cough in 30.4%, breathing difficulty in 29.3%, pain in 26.8%, and insomnia in 28.3%; (b) psychological issues of fear in 27%, worry in 27.3%, depression in 16.6%, and anger in 17.7% (Figure 2). An in-depth comparison of physical and psychological severity scores (“Quite a Bit” and “Very Much”), as per gender, hospitalization, and age (<50/>50 years) revealed the following: (a) females suffered certain physical symptoms, namely fatigue (p=0.016), nausea (p=0.039), and constipation (p=0.05); (b) cough and breathing difficulty were higher in the non-hospitalized group (22.6% versus 34.2%; p=0.077); (c) severity of psychological symptoms showed no difference in terms of gender, whether the patient was hospitalization or not, and the age of the patient (<50/>50 years).

We recorded the socioeconomic conditions before and after COVID-19 infection, in order to compare the impact on the patients and their families (Table 2). For a median of 37 days (minimum 14 days, maximum 141 days) from the time of COVID-19 positive status, the employment status was documented for 188 out of 198 patients as employed (salaried), self-employed, and retired in 45.6%, 16.6%, and 12.4%, respectively. The median monthly income in Indian Rupees (INR) for pre-Covid versus post-Covid was 30,000 versus 25,000, showing a loss of 16.6% in family income in post-COVID-19 conditions. The households of these PCC surveyed persons showed a picture of total family members at a median of 4 (range: 2 to 13) and the number of dependents at a median of 3 (range: 0 to 12). Out-of-pocket expenditures (OOPE) for the COVID-19 diagnosis and treatments was recorded for 153 (77%) patients out of the entire cohort, for a median OOPE of 9000 (0 to1300000) INR, and 41 persons (20%) had lost their entire income post Covid.

**Table 2:** Socioeconomic Analysis Post the COVID-19 Survey

Variable	Number of Patients surveyed	Median (Min-Max)		
Total Patients(included in the study)	198	-		
Time (in days) between date of diagnosis and post-Covid survey)	198	37 (14-141)		
*Employment Status^1^	188*	-		
Monthly Income-Pre-Covid(Indian Rupees,INR)	164	30000 (0-800000)		
Post-Covid Monthly Income(Indian Rupees,INR)	154	25000 (0-200000)		
No. Dependents	140	3 (0-12)		
Monthly House Expenditure(INR)	161	20000 (0-1000000)		
Other Earnings in Household per Month(INR)	66	19000 (0-4000000)		
Total Family Members in House	143	4 (2-13)		
Out-of-Pocket expenditure (in INR for Covid-19 diagnosis and treatments)	157	9000 (0-1300000)		

**Figure 3:**
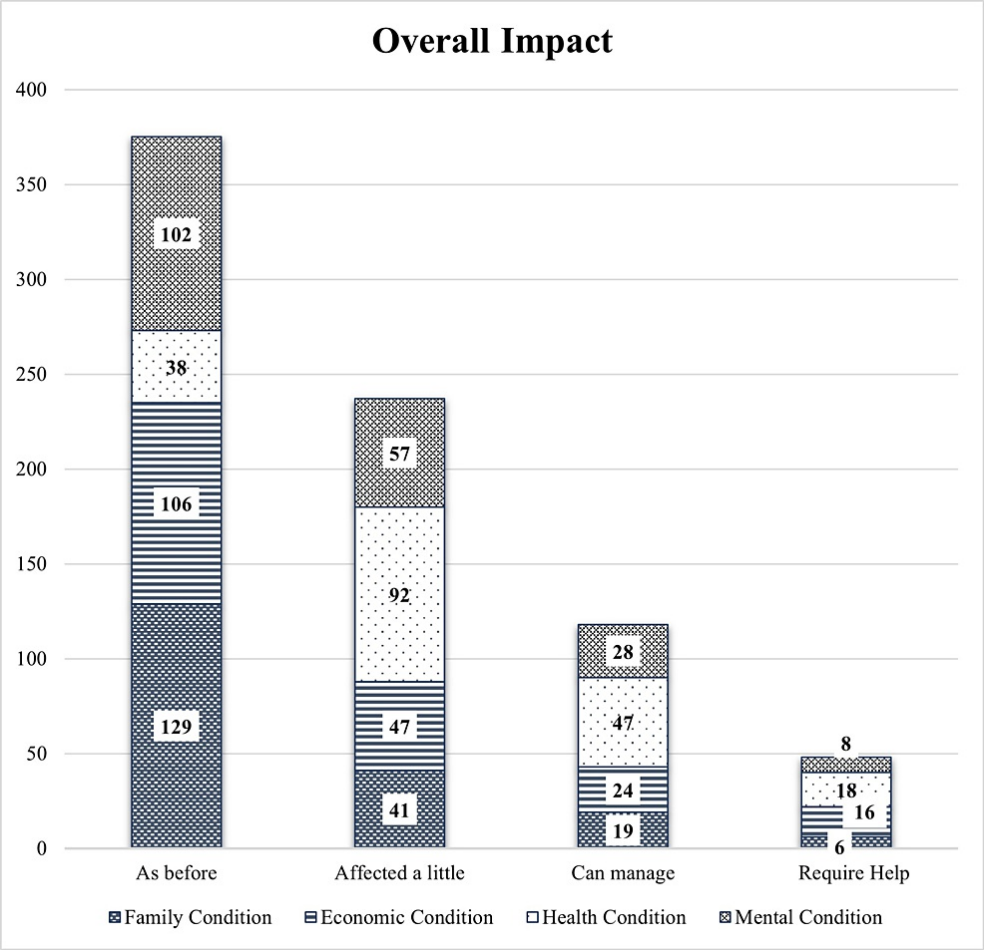
Overall Impact

The overall impact of COVID-19 is shown in Figure 3. Overall, 31.3% and 20.7% had an adverse impact on their health and economic conditions.

## Discussion

This report from Odisha has attempted a prospective cross-sectional survey of the physical and psychological symptoms and socioeconomic impact experienced by post-COVID-19 recovered persons. To our knowledge, this is the first report on this topic from this region of India aside from another published paper from North India [10]. 

Many studies have been carried out to model the COVID-19 pandemic in India, including the recovery from the acute phase of infection. A nationwide study has shown that the average time of the infectious phase ranged from five days to 68 days. In Odisha, the acute COVID-19-positive stage, where a person remained infected, was an average of 13 days (range: 11 to 15 days) [11]. We also have to take into consideration that most Indian states have different situations concerning the COVID-19 pandemic and have unique problems to tackle.

Country-wise incidence burden of COVID-19 has shown that India is ranked second to the USA [1]. After the infective phase, the clinical status of the recovered post-COVID-19 persons can have implications based on the socio-geographic and healthcare conditions. Health systems in developed countries were quick to evaluate the long COVID or PCC trajectory for those with prolonged disease conditions [2-7]; however, the overwhelming majority of low- and middle-income (LMIC) countries still lack the manpower, infrastructure, and research [12]. The available published papers from India, instead of covering the overall effects on the post-COVID-19 status, have reported specific post-COVID-19 situations like mucormycosis [14], heart rate variability [15], pulmonary fibrosis [16], gastrointestinal disorders [17], and impact of the pandemic on health workers [18]. 

In the USA, Europe, and China, the persistent physical and psychological symptoms of recovered persons, beyond four weeks of COVID-19 diagnosis, show a prevalence of 10% to around 80% [19,20]. A comprehensive understanding of patient care beyond the acute phase will help in the development of infrastructure for COVID-19 clinics that will be equipped to provide integrated multispecialty care in the outpatient setting [19]. The post-COVID-19 trajectory, when closely followed in recovered persons, has recorded certain key findings: a. more than 25% show new-onset symptoms that were not present at the acute phase; b. the impact of the disease on patients' lives began increasing six months after onset; c. the sequelae of episodic increase in symptoms may require hospitalization for nearly 15% [19,21,22] of the population. All these emerging features of long COVID or PCC can have long-term implications for health systems.

Facing the demand from self-referred patients who had lingering and multiple symptoms, the Mayo Clinic, Rochester, Minnesota, USA set up a multi-disciplinary COVID-19 Activity Rehabilitation Program (CARP), with three objectives: evaluate associated conditions in the recovery phase, facilitate improvement in function, and promote a therapeutic and safe pathway for return to work or normal activities [23]. In line with the similar healthcare needs of the patients who were discharged from the Covid hospital set up within this academic medical institution, the post-Covid clinic was managed by physicians with nurses and paramedics. From a cross-sectional study, a proportion of patients attending this clinic showed five dominant lingering and bothersome physical symptoms viz. fatigue (82.8%), cough (54%), breathing difficulty (54%), pain in the body (53%), and sleeplessness (51%). Additionally, fear (41.6%), worry (40.4%), depression (31.8%) and anger (30.3%) were the prolonged psychological effects. Hence, 50% of those surveyed experienced more than one physical symptom, and 40% carried one or more signs of psychological distress beyond four weeks of COVID-19 diagnosis. The post-Covid clinic survey from the USA [23], with a similar median age of 45 years and follow-up for at least four weeks or more after confirmed COVID-19 infection, has shown presentations of fatigue (80%), followed by respiratory complaints (59%) and neurological complaints (59%).

Although the knowledge and clinical management of PCC are evolving, the published reviews have substantial datasets on physical and psychosocial symptom burdens [3,19,20], which corroborate the original prospective and retrospective studies [2,7,21,23]. Overall, the information for post-COVID-19 conditions can be summarized as follows: 1. persistent symptoms seen in 10% to above 80%; 2. more than one symptom observed in 30% to 80%; 3. new-onset symptoms and increased impact observed beyond six months; 4. fatigue, cough, respiratory distress, pain, and insomnia are the main physical symptoms in a range of 10% to 80%; 5. anxiety/depression, fear, and worry are psychological issues faced by 10% to 40%.

In our present management strategies, it is not routinely recommended to carry out tests unless the patient has a specific condition related to the pulmonary, cardiac, renal, neurologic, or gastrointestinal system(s). Age above 65 years, female gender, and associated comorbidities can increase the symptom burden, and prolong the recovery time. In addition, there is a need for hospital admission and management in approximately 15% of those with PCC [3,4,7,19,20].

Besides the physical, psychological, and medical care, the socioeconomic assessment of the PCC persons living with long COVID/PCC should be a major consideration for health policymakers and healthcare providers. Our cross-sectional study has shown that for an average family of four members with three dependents, the monthly income was INR 30,000 (USD 405) in pre-Covid. This monthly income showed a decrease to INR 25,000 (USD 337.5); effectively a family’s purchasing power had reduced by 16.7% at the post-Covid level. The post-Covid survivors had spent a median of INR 9,000 (USD 121.5) for COVID-19 treatment which is approximately 30% of their monthly income.

Even though the national and state governments in India have made substantive plans on public health expenditures for diagnosis, treatment, hospitalization, vaccination, etc., the OOPE by the infected COVID-19 persons can cause an economic burden on families. Our findings from Odisha have many similarities to the neighboring state of Chhattisgarh, as both states have identical GDP per capita. A primary survey of COVID-19 treatment in Chhattisgarh calculated the mean OOPE on hospitalization as INR 169,504 (142,094-196,914) in private hospitals and INR 4,871 (3068-6674) in public hospitals [24]. Another study on COVID-19 patients from Western India revealed that even after the government scheme, the median difference in the final bill and the amount reimbursed through government schemes was INR 59,560 ($ 807.75) [25]. Thus a mix of limited published literature has quantified substantial OOPE borne by the COVID-19 infected patients for accessing healthcare services in India. Populations LMICs have been disproportionately affected by the pandemic, which brought about an additional burden on their already weak health systems [26]. To address the socioeconomic impact of the COVID-19 pandemic and its after-effects on the vulnerable population, the health expenditures done as OOPE should be monitored by healthcare providers, health policymakers, and the governments in LMICs.

The global COVID-19 pandemic has necessitated rapid responses to prevent transmission, manage acute/infective phases, and provide coverage for vaccination. Yet, it is recognized that the long-term health consequences of COVID-19 remain unknown, and there is a significant gap in conducting research related to the core outcome sets for post-COVID-19 conditions [27]. The WHO has designed CRF for PCCs which includes three modules: Module 1 includes background demographic and clinical information of the acute episode of COVID-19. Module 2 includes questions to help identify patients who require further clinical evaluation. Module 3 includes medical assessment and results of examinations, tests, or diagnoses made during the follow-up visit. Subsequently, based on the results, patients should be referred for clinical care or rehabilitation as per national protocols [28].

The limitation of this study was to evaluate the post-COVID-19 condition of the patients captured as cross-sectional data for subsequent analysis. Hence, in the absence of a temporal time trend, the changes in physical and psychological symptom burden or the dynamics of socio-economic conditions could not be recorded for any comparison. However, from the available cross-sectional data, the benefits of the post-Covid clinic for the recovery and rehabilitation of COVID-19 infected individuals would require further implementation by the public health policy measures.

## Conclusions

This clinical cross-sectional study from Odisha, where the socioeconomic condition of the post-COVID-19 recovered persons have a low GDP per capita, revealed persistent physical and psychological symptom burdens and its consequent financial impact on the families. Due to the inadequacy of available healthcare resources, the recognition of the post-COVID-19 conditions in LMICs would help mitigate the needs of the local population. The post-Covid clinic can be a resource-appropriate health system approach for the pandemic survivors. At a modest estimate of 20% of the recovered persons in post-COVID-19 conditions seeking medical care long term, approximately 8 million will be treated promptly; They will be provided designated post-Covid clinics across the country, at 727 districts in India, for at least the upcoming two years. The median age of 41 years makes it imperative that accessible medical care and rehabilitation can encourage the return to socioeconomic balance. This can augment the future preparedness for the increasing recurrences of infectious diseases which cross borders in a globally interconnected world.
